# Pharmacokinetic modelling of MRI-based liver function for risk assessment in primary sclerosing cholangitis: a prospective pilot study

**DOI:** 10.1186/s41747-026-00764-5

**Published:** 2026-06-18

**Authors:** Wolf C. Bartholomä, Shan Cai, Christian Simonsson, Markus Karlsson, Stergios Kechagias, Mischa Woisetschläger, Nils Dahlström, Peter Lundberg

**Affiliations:** 1 Centre for Medical Image Science and Visualisation (CMIV), Linköping University, Linköping, Sweden; 2 Department of Medical and Health Sciences, Linköping University, Linköping, Sweden; 3 Clinical Department of Radiology in Linköping, Region Östergötland, Linköping, Sweden; 4 Department of Biomedical Engineering, Linköping University, Linköping, Sweden; 5 Clinical Department of Gastroenterology and Hepatology, Region Östergötland, Linköping, Sweden; 6 Clinical Department of Medical Radiation Physics, Region Östergötland, Linköping, Sweden

**Keywords:** Cholangitis (sclerosing), Disease progression, End stage liver disease, Magnetic resonance imaging, Prognosis

## Abstract

**Objective:**

Primary sclerosing cholangitis (PSC) is a rare fibroinflammatory hepatobiliary disease with a highly variable clinical course. Identifying patients at risk for poor outcomes remains challenging. Magnetic resonance imaging (MRI)-based approaches such as DiStrict, Anali score, and relative enhancement (RE) show promise but are limited by operator dependency or static measurements. This study explored pharmacokinetic modelling of liver function as a quantitative imaging biomarker for risk assessment in PSC.

**Materials and methods:**

A prospective cohort of 26 PSC patients underwent up to five annual MRI examinations with follow-up up to 7.5 years. Clinical endpoints included liver transplantation, decompensated cirrhosis, and cholangiocarcinoma. Correlation and receiver operating characteristics (ROC) analyses compared the pharmacokinetic model with Anali scores, RE, model for end-stage liver disease (MELD), and the Amsterdam–Oxford Model (AOM).

**Results:**

The pharmacokinetic model (*k*_single_) correlated significantly with MELD (*r* = -0.429, *p* = 0.029), AOM (*r* = -0.557, *p* = 0.003), and endpoint events (*r* = -0.605, *p* = 0.001). ROC analysis showed excellent discrimination for *k*_*i,single*_ (area under the curve [AUC] = 0.943) outperformed Anali scores (AUC = 0.800–0.829) and comparable to MELD (AUC = 0.857) and AOM (AUC = 0.900).

**Conclusion:**

Pharmacokinetic liver function modelling correlated strongly with MELD and AOM, effectively identifying high-risk PSC patients.

**Relevance statement:**

Pharmacokinetic liver function modelling detects functional impairment in PSC, correlating well with established tools such as the AOM. As an objective, quantitative imaging biomarker, this method may complement established risk scores and aid in the identification of patients at risk of adverse outcomes.

**Key Points:**

- Pharmacokinetic modelling estimates changes in liver function based on MRI.
- These estimates can be used as a prognostic tool in PSC.
- The model’s prognostic performance was comparable to established clinical tests.

**Graphical Abstract:**

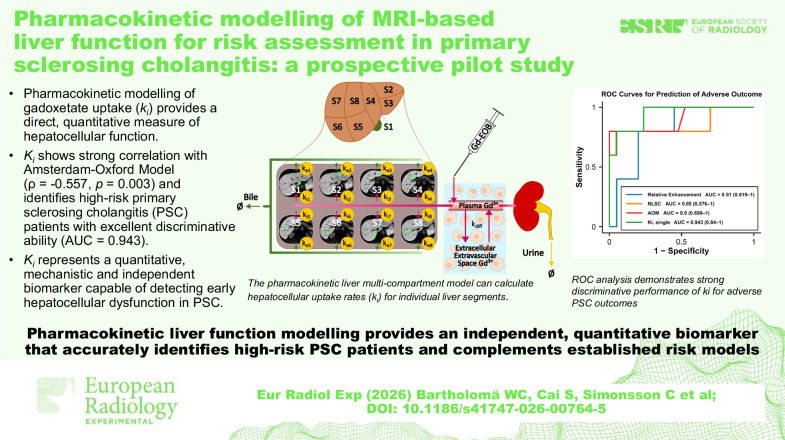

## Background

Primary sclerosing cholangitis (PSC) is a rare, idiopathic, fibroinflammatory disease of the biliary system, characterised by inflammation, fibrosis, and multifocal stricturing of the intra- and extra-hepatic bile ducts [1]. It causes progressive liver damage that can lead to cirrhosis, portal hypertension, and end-stage liver disease, with liver transplantation being the only definitive treatment [2]. PSC carries an increased risk of hepatobiliary malignancies such as cholangiocarcinoma, hepatocellular carcinoma, and gallbladder carcinoma. The clinical course of PSC is highly variable, ranging from mild and largely asymptomatic disease to severe, rapidly progressive forms requiring liver transplantation, sometimes at an early age [3].

Identifying patients at risk of poor prognosis is essential but remains challenging [4], as there is a lack of consensus on an optimal method for risk assessment. Current guidelines advocate a multimodal approach that combines:

- biochemical markers such as alkaline phosphatase, serum bilirubin, albumin, aspartate aminotransferase, alanine aminotransferase, platelet count, and CA 19-9;
- risk scores and prognostic models, including the Mayo risk score, model for end-stage liver disease (MELD), and the Amsterdam–Oxford Model (AOM); and
- non-invasive imaging tests such as transient elastography (FibroScan®), magnetic resonance elastography, and magnetic resonance imaging (MRI) with magnetic resonance cholangiopancreatography [5, 6].

The value of MRI and magnetic resonance cholangiopancreatography in PSC lies primarily in cholangiocarcinoma surveillance. Its utility in predicting liver function decline and overall prognosis is less well established. Non-standardised, subjective assessments of liver and bile duct morphology alone have shown limited prognostic value [7]. Consequently, several structured and standardised imaging approaches have been developed, including the DiStrict score [7], which is based on morphological bile duct changes, and the Anali scores, which combine morphological and contrast-derived features [8]. Functional approaches, such as relative enhancement (RE), quantify hepatobiliary contrast uptake [9]. However, although each method has shown promise, they remain limited by significant operator dependency, as in DiStrict or Anali scores [7, 10], or by reliance on single-phase contrast measurements [9] that fail to reflect dynamic liver function.

A whole-body pharmacokinetic (PK) liver function model based on hepatobiliary contrast uptake across multiple dynamic time points offers a structured, objective, and fully quantitative means of directly assessing liver function. This model has been validated in both healthy individuals and patients with chronic liver disease and has been reported to detect early reductions in hepatocellular function [11–13].

This pilot study aimed to evaluate whether the hepatocellular functional information provided by this PK liver function model could serve as a prognostic biomarker in PSC and to compare its performance to structural MRI-derived scoring systems (Anali scores), other quantitative MRI approaches (*i.e*., RE), and established clinical risk scores (AOM, MELD).

## Methods

### Patients

This study consecutively included patients with PSC from a single centre, Linköping University Hospital, who were part of a Swedish multicentre prospective surveillance study on PSC, enroled from 2011 and followed up until 2021. The study was approved by the Institutional Review Board (Dnr 2011/824-31/2, 2018/1111-32, and 2018/1494-31/3). Written informed consent was acquired from each patient. A total of 33 patients were enroled. Each patient underwent up to five annual serial MRI examinations with hepatobiliary contrast, including magnetic resonance cholangiopancreatography, along with long-term clinical follow-up. For the present analysis, the last available MRI examination during follow-up was used for each patient.

### Laboratory and clinical data

Relevant laboratory and clinical data were extracted and pseudonymised by a hepatologist from the Electronic Patient Record (Cosmic, Cambio Healthcare Systems AB). MELD scores and AOM values were calculated based on the clinical data. All laboratory values were from the same time point as the evaluated imaging data.

### MRI

Imaging was acquired on a single 1.5-T scanner (Achieva, Philips Medical Systems) using a phased-array body coil. Patients were instructed to fast for four hours before the examination. Hyoscine butylbromide (Buscopan™, Sanofi S.r.l.) was administered to reduce gastrointestinal motility (1 mL intravenously and 1 mL intramuscularly).

Gadoxetate-enhanced images were acquired using a breath-hold, fat-saturated, T1-weighted three-dimensional gradient-echo sequence. Typical imaging parameters included: flip angle = 10°; repetition time = 4.2 ms; echo time = 2.0 ms; sensitivity encoding factor = 1.7; and acquisition volume = 300 × 200 × 350 mm³. Images were acquired before and after intravenous bolus injection of gadoxetate (gadolinium-ethoxybenzyl-diethylenetriamine pentaacetic acid [Gd-EOB-DTPA], Primovist/Eovist®, Bayer Schering Pharma) at a dose of 0.025 mmol/kg body weight. Post-injection images were obtained at multiple contrast phases, including arterial and portal venous phases, with transitional phase imaging at 3 and 5 min and hepatobiliary phase imaging at 10 and 20 min and beyond, including additional time points up to 43 min.

### Image postprocessing

Images were exported from the Picture Archiving and Communication System (Sectra Imtec AB) and analysed using Mathematica (Wolfram Research Inc.). Regions of interest (ROIs) were placed by two abdominal radiologists with experience in hepatobiliary MRI who were blinded to patient status and pathology at the time of ROI placement. To minimise reader-related bias, half of the patients were initially analysed by each reader. For each examination, one freehand ROI was placed in each Couinaud liver segment, avoiding visible vascular structures and bile ducts. In addition, three splenic ROIs (superior, perihilar, and inferior) were placed centrally within the spleen, maintaining an approximate distance of 1 cm from the splenic capsule.

**Fig. 1:**
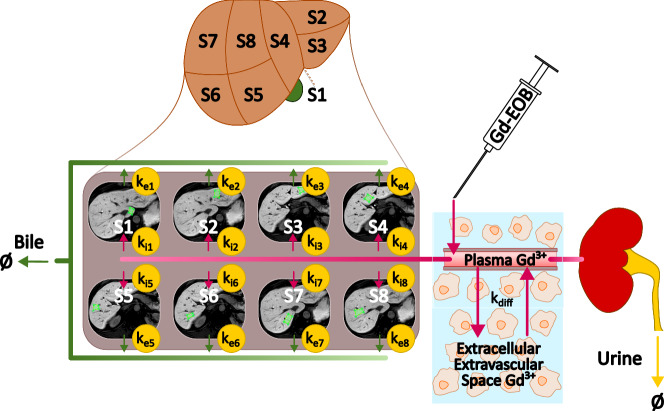
Schematic for the segmental liver PK model. The global liver PK model works similarly but treats the entire liver as one single compartment instead. *Gd-EOB-DTPA* Gadolinium-ethoxybenzyl-diethylenetriamine pentaacetic acid (Gadoxetate, Primovist), k_diff_ Rate of diffusion between plasma and extracellular extravascular space, *k*_*i1*_*-k*_*i8*_ Influx (intracellular uptake rate) into liver segments S1 to S8, *S1–S8* Couinaud liver segments (segment 4 is treated as one unit, not subdivided into 4A and 4B). The model states represent the concentration of Gd-EOB-DTPA in each of the eight individual S1 to S8 Couinaud segments. In the model, each segment is defined by a compartment volume, as well as individual influx *k*_*i*_ and efflux *k*_*e*_ rate parameters. The Gd-EOB-DTPA concentration present in plasma is described in the Plasma Gd compartment, and the concentration in the extracellular extravascular space is defined by the ECS Gd compartment. The rate of diffusion between the plasma and extracellular extravascular space is defined by the rate parameter k_diff_. The clearance of Gd-EOB-DTPA via urine is fixed at a constant rate. The model parameters were estimated so that the model simulations agreed with the measured concentration (converted from signal intensity) for each segment in each time point. The model can produce a simultaneous agreement for all segments, as well as data from the spleen

After initial placement, all ROIs were reviewed jointly by both readers and adjusted where necessary in consensus. Final consensus ROIs were propagated automatically to all corresponding time points, anatomically matched, and adjusted for motion. Across all liver segments except segment 1, the mean ROI size was 251 ± 99 mm^2^ (mean ± standard deviation). Due to the physiologically smaller size of segment 1, the average ROI size was smaller at 189 ± 94 mm^2^. The mean splenic ROI size was larger and more variable (612 ± 554 mm^2^), reflecting physiological and disease-related differences in splenic size. An example of ROI size and placement in the liver is shown in Fig. 1.

### PK modelling

The modelling approach has been previously described [11–13]. In this study, two variations of the PK liver function model were used: the validated global liver PK model and a derived segmental liver PK model allowing estimation of contrast agent dynamics in each Couinaud liver segment. Briefly, both the global and segmental PK liver models were constructed using ordinary differential equations (ODEs) and implemented in MATLAB (2022a, MathWorks) using the IQMtoolbox package [14]. The full model description can be found in Supplementary Material Section 1 – Mathematical modelling, and a model schematic of the segmental liver PK model is shown in Fig. 1. All code for the model analysis can be found in our GitHub Repository (https://github.com/chrsi30/psc). Both models were used to estimate the hepatobiliary contrast uptake rate (*k*_*i*_, s⁻¹) by finding agreement between the model simulation of gadoxetate concentration in the liver and spleen, and measured values. The parameter estimation was carried out using the enhanced scatter search algorithm from the MEIGO toolbox [15]. All model simulations demonstrated satisfactory agreement with the data (for examples of model agreement, see Supplementary Material Section 1—Figs. S1 and S2).

Global liver function was either directly derived from the global liver PK model (*k*_*i,single*_) or calculated as the mean value of the hepatic uptake rate (*k*_*i,multi*_) across all segments (*k*_*i1*_–*k*_*i8*_) in the segmental liver PK model.

### RE and normalised liver-spleen contrast ratio (NLSC)

RE was calculated from the mean signal intensities of all liver segments, obtained pre-contrast (SI_liverpre_) and at the 20-min hepatobiliary phase (SI_liver20min_), according to the formula:

$${RE}=\frac{{{SI}}_{{liver}20\min }-{{SI}}_{{liverpre}}}{{{SI}}_{{liverpre}}}\times 100 \%$$

as described by Schulze et al [9].

NLSC was calculated using the mean liver and spleen signal intensities pre-contrast (SI_liverpre_, SI_spleenpre_) and at the 20-min hepatobiliary phase (SI_liver20min,_ SI_spleen20min_):

$${NLSC}=\frac{{{SI}}_{{liver}20\min }/{{SI}}_{{liverpre}}}{{{SI}}_{{spleen}20\min }/{{SI}}_{{spleenpre}}}$$

as proposed by Dahlqvist Leinhard et al [11].

The ROIs applied for calculating RE and NLSC were the same as for the PK model.

### Anali scores

ROI placement for the PK analysis was performed first. Anali scores were subsequently evaluated by an abdominal radiologist with 15 years of experience, blinded to all clinical data except for the diagnosis of PSC, using the same MRI examinations as those used for the ROI-based PK analysis. This radiologist had previously participated in ROI placement for approximately half of the patients and in the subsequent consensus review of all ROIs; however, Anali's assessment was conducted with a temporal separation of approximately two years from the ROI-based analysis, which is expected to substantially reduce recall-related bias. The radiologist was blind to the endpoint-event status of each patient.

For each patient, intrahepatic bile duct dilatation was assessed in the peripheral intrahepatic biliary tree, with the most dilated duct scored as 0 if the duct diameter was ≤ 3 mm, 1 if 4 mm, and 2 if ≥ 5 mm.

Liver deformity (atrophy, lobulated liver surface, increased caudate-to-right-lobe ratio), portal hypertension (portosystemic collaterals and/or splenomegaly), and parenchymal enhancement heterogeneity (in arterial and hepatobiliary phases) were each scored 0 = absent and 1 = present.

The three different Anali scores were calculated as proposed by Ruiz et al [8]:

- Anali score based on morphology alone (Anali NoGd) = (1 × intrahepatic bile duct dilatation) + (2 × liver deformity) + (1 × portal hypertension);
- Anali score based on deformity and the arterial enhancement pattern (Anali GdAP) = (1 × liver deformity) + (1 × enhancement heterogeneity in the arterial phase);
- Anali score based on deformity and hepatobiliary enhancement pattern (Anali GdHBP) = (1 × liver deformity) + (1 × enhancement heterogeneity in the hepatobiliary phase).

### Outcomes

Primary endpoint outcomes included decompensated liver cirrhosis (with implantation of a transjugular portosystemic shunt counted as evidence of decompensation), liver transplantation, hepatocellular cancer, and liver-related death.

### Statistical analysis

This study was designed as an exploratory biomarker evaluation and proof-of-concept investigation rather than the development of a formal prognostic prediction model. Therefore, analyses focused on correlation and discriminative performance rather than time-to-event modelling.

Statistical analyses were conducted using SPSS (IBM Corp.), R (version 4.5.1; R Foundation for Statistical Computing), or MATLAB (2024a, MathWorks). A *p*-value < 0.05 was considered statistically significant. Demographic, laboratory, and clinical data corresponding to the last available MRI examination were summarised descriptively. Continuous variables were expressed as median and interquartile range, and categorical variables as counts. For variables with missing data, the variables were calculated on the basis of the available data.

The normality of variables (*e.g*., Anali NoGd, Anali GdAP, Anali GdHBP, *k*_*i,single*_, *k*_*i,multi*_) was assessed using the Shapiro–Wilk test. Univariate analysis of statistical differences was performed using the Mann–Whitney *U*-test, and correlations and associations were assessed using Spearman’s rank correlation coefficient (*ρ*), χ^2^ tests, and coefficient of variation as appropriate.

Receiver operating characteristic (ROC) curve analysis and DeLong tests were used to evaluate the predictive performance of Anali NoGd, Anali GdAP, Anali GdHBP, RE, NLSC, *k*_*i,single*_, *k*_*i,multi*_, MELD, and the AOM for endpoint events.

To further investigate the dominant patterns or sources of variability in *k*_*i,multi*_, a principal component analysis was conducted on the standardised (z-scored) segmental PK parameters (*k*_*i1*_–*k*_*i8*_).

### Language editing

ChatGPT has been used for language editing prior to submission for proofreading by a language editing service (Anchor English, English correction and proofreading service). The authors remain fully responsible for all content.

## Results

### Study population

**Fig. 2:**
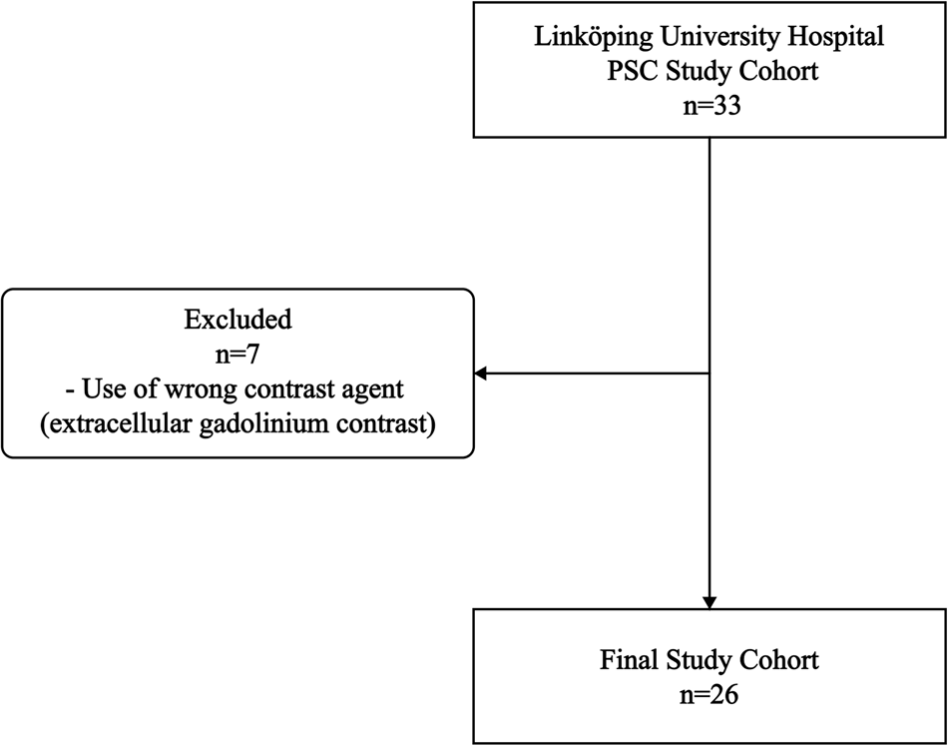
Study flowchart

Of the 33 patients enroled, a total of 26 patients were included in the analysis (Fig. 2). Seventeen patients were male, and nine were female, with a median age of 41 years (range 37–53 years). Twenty-four patients had large duct PSC, and the remaining two had small duct PSC. The median age at disease presentation was 31 years (range 15–63 years). The disease stage and risk were predominantly moderate, reflected by a median MELD score of 6 (range 6–14) and an AOM score of 1.58 (range 0.86–4.08).

Follow-up time for endpoint events was up to 7.5 years. No endpoint event was observed in patients with small duct PSC. One individual suffered from multiple endpoint events (cholangiocarcinoma and liver-related death). This was counted as only one endpoint event. The median time between the last MRI examination performed and an endpoint event was 1,326 days (range 728–2,078 days). Detailed study population characteristics, including laboratory values, are provided in Table 1.

**Table 1:** Patient characteristics

Characteristic	Values	Normal range
Patients (number of)	26	−
Male/female	17/9	−
Age (years)	41 (14.8)	−
Age at PSC diagnosis (years)	31 (16.2)	−
PSC subtype	Large duct (*n* = 24), small duct (*n* = 2)	−
ALT (µkat/L)	0.70 (1.10)	< 0.76
AST (µkat/L)	0.61 (0.86)	< 0.61
ALP(µkat/L)	2.20 (4.5)	0.70–1.90
γGT (µkat/L)	2.70 (6.3)	< 1.3
Total serum bilirubin (µmol/L)	11 (15)	< 26
Albumin (g/L)	39 (6)	34–45
Platelets (x10^9^/L)	226 (134)	150–450
Creatinine (µmol/L)	74 (21)	45–90
INR	1.0 (0.1)	0.8–1.2
MELD score	6 (8)	−
AOM	1.58 (3.22)^*^	−
Number of endpoint events	5	−
Type of endpoint event:		
Liver transplatation	3	−
TIPS	1	−
Cholangiocarcinoma	1	−
Days between last MRI and Endpoint Event	1326 (728−2078)	−
Death	1	−

Laboratory values and clinical scores at the time of the last recorded MRI examination. Medians are calculated. Interquartile range is given in parentheses apart from endpoint event, where ranges are given. No multiple endpoint events occurred in the same individual

*Age* Age at last MRI examination, *ALP* Alkaline phosphatase, *ALT* Alanine transaminase, *AST* Aspartate transaminase, *γGT* Gamma-glutamyl transferase, *INR* International normalised ratio, *MELD* Model for end-stage liver disease, *MRI* Magnetic resonance imaging, *PSC* Primary sclerosing cholangitis, *TIPS* Transjugular intrahepatic portosystemic shunt

^*^AOM is validated only for adult-onset PSC. One patient was 15 at PSC presentation—for the calculation of the AOM score in this patient, an age of 18 was assumed

### Comparison with imaging-based scores: Anali

All Anali scores showed moderate positive correlations with AOM scores (*ρ* = 0.490–0.532, *p* = 0.005–0.011) and endpoint events (*ρ* = 0.417–0.478, *p* = 0.014–0.034). Anali NoGd and GdHBP also correlated moderately to strongly with MELD scores (*ρ* = 0.419–0.594, *p* = 0.001–0.033). Both Anali NoGd and GdHBP correlated moderately with bilirubin (*ρ* = 0.487–0.566, *p* = 0.003–0.012), while Anali NoGd correlated moderately with γGT (*ρ* = 0.446, *p* = 0.022) and Anali GdAP with platelets (*ρ* = 0.405, *p* = 0.040) and INR (*ρ* = 0.403, *p* = 0.041).

### Comparison with static hepatobiliary metrics: RE and NLSC

RE was negatively correlated with both MELD and AOM (*ρ* = -0.497, *p* = 0.011; *ρ* = -0.585, *p* = 0.002, respectively), while NLSC exhibited strong negative correlations (MELD: *ρ* = -0.714, *p* < 0.001; AOM: *ρ* = -0.639, *p* < 0.001). Both RE and NLSC showed negative correlations with endpoint events (RE: *ρ* = -0.430, *p* = 0.032; NLSC: *ρ* = -0.485, *p* = 0.014). For laboratory values, RE was negatively correlated with bilirubin (*ρ* = -0.618, *p* < 0.001) and positively correlated with platelets (*ρ* = 0.525, *p* = 0.008) and creatinine (*ρ* = 0.421, *p* = 0.036). NLSC demonstrated a negative correlation with γGT (*ρ* = -0.426, *p* = 0.034), a strong negative correlation with bilirubin (*ρ* = -0.706, *p* < 0.001), and a positive correlation with platelets (*ρ* = 0.529, *p* = 0.008).

### Primary PK model parameters

Very strong correlations were seen between *k*_*i,single*_ and *k*_*i,multi*_ (*ρ* = -0.878, *p* < 0.001), and neither showed significant correlations with laboratory values apart from platelets (*ρ* = 0.614, *p* = 0.001 for *k*_*i,single*_, and *ρ* = 0.489, *p* = 0.012 for *k*_*i,multi*_). *K*_*i,multi*_ had weak negative correlations with MELD (*ρ* = -0.393, *p* = 0.047) and moderate negative correlations with AOM and endpoint events (*ρ* = -0.490, *p* = 0.011; *ρ* = -0.514, *p* = 0.007). *K*_*i,single*_ showed moderate negative correlations with MELD and AOM (*ρ* = -0.429, *p* = 0.029; *ρ* = -0.557, *p* = 0.003) and correlated strongly with endpoint events (*ρ* = -0.605, *p* = 0.001).

**Fig. 3:**
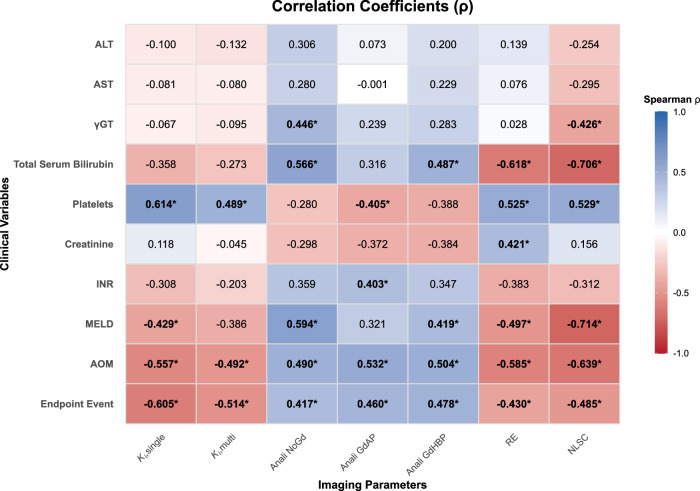
Correlation of prognostic parameters with laboratory data, clinical scores, and endpoint events. All values represent Spearman correlation coefficients (*ρ*). Significant correlations are shown in bold and marked with an asterisk (*). Positive correlations are displayed in blue and negative correlations in red, with increasing colour intensity indicating higher correlation strength. *ALP* Alkaline phosphatase, *ALT* Alanine transaminase, *Anali GdAP* Anali score based on gadolinium contrast features in the arterial phase, *Anali GdHBP* Anali score based on gadolinium contrast features in the hepatobiliary phase, *Anali NoGd* Anali score derived without gadolinium contrast, *AOM* Amsterdam–Oxford model, *AST* Aspartate transaminase, *γGT* Gamma-glutamyl transferase, *INR* International normalised ratio, *MELD* Model for end-stage liver disease, *NLSC* Normalised liver–spleen contrast ratio in the hepatobiliary phase (20 min), *RE* Relative liver enhancement in the hepatobiliary phase (20 min), *k*_*i,single*_ Hepatocellular uptake rate based on the global liver PK model, *k*_*i,multi*_ Hepatocellular uptake rate based on the segmental liver PK model

Correlation coefficients, *p*-values, and non-significant findings (including alanine aminotransferase, aspartate aminotransferase, albumin, and creatinine) are visualised in Fig. 3. All correlation results in table form, including exact *p*-values, are provided in Supplementary Table S1.

### Comparison of prognostic parameters between patients with and without endpoint events

All evaluated parameters showed statistically significant differences between patients with and without endpoint events. Further details can be found in Table 2.

**Table 2:** Comparison and statistical differences between patients with and without endpoint events

	No endpoint event (*n* = 21)	Endpoint event (*n* = 5)	*p*-values
*k*_i, single_ (s^-1^)	0.0028 (0.0036)	0.0005 (0.0017)	< 0.001
*k*_i, multi_ (s^-1^)	0.0033 (0.0026)	0.0009 (0.0019)	0.008
Anali NoGd	1 (5)	4 (2)	0.041
Anali GdAP	1 (2)	2 (1)	0.028
Anali GdHBP	1 (2)	2 (1)	0.023
RE^*^ (%)	87.51 (143.06)	66.13 (39.03)	0.035
NLSC^*^	1.50 (0.20)	1.13 (0.33)	0.015
MELD	6 (4)	11 (8)	0.012
AOM	1.48 (1.68)	3.38 (2.60)	0.003

Median values are given for each subgroup, with interquartile ranges in parentheses. To test for statistically significant differences, the Mann–Whitney *U*-test was used. *p*-Values calculated at exact significance levels because of small sample size

*Anali GdAP* Anali score based on gadolinium contrast features in the arterial phase, *Anali GdHBP* Anali score based on gadolinium contrast features in the hepatobiliary phase, *Anali NoGd* Anali Score derived without gadolinium contrast, *AOM* Amsterdam–Oxford model, *k*_*i,multi*_ Hepatocellular uptake rate based on the segmental liver PK model, *k*_*i,single*_ Hepatocellular uptake rate based on the global liver PK model, *MELD* model for end-stage liver disease, *NLSC* Normalised liver–spleen contrast ratio in the hepatobiliary phase (20 min), *RE* Relative liver enhancement in the hepatobiliary phase (20 min)

^*^ Missing data: Data missing from one patient without an endpoint event

### ROC analysis of prognostic parameters

In ROC analysis, Anali NoGd had an area under the curve (AUC) of 0.800, while Anali GdAP and Anali GdHBP scored slightly higher at 0.814 and 0.829, respectively. RE and NLSC also performed well, with AUCs of 0.810 and 0.850, respectively. AOM and MELD had AUCs of 0.900 and 0.857, respectively. *K*_*i,single*_ and *K*_*i,multi*_ outperformed the Anali scores, RE, NLSC, and MELD, and in the case of *K*_*i,single*_, even AOM, achieving AUCs of 0.943 and 0.876, respectively. The largest AUC difference, seen between Anali NoGd and *k*_*i,single*_, was 0.143, though this was not statistically significant (DeLong test, *p* = 0.061). All AUCs, *p*-values, and 95% CIs are detailed in Table 3. ROC curves are provided in Supplementary Fig. S3. Sensitivities and specificities are provided in Supplementary Table S2.

**Table 3:** Areas under the curve (AUC) from ROC analysis of *Ki,single*, *Ki,multi*, RE, NLSC, Anali scores, MELD, and AOM for endpoint events

Parameter	AUC	*p*-values	95% CI
*k*_*i, single*_ (s^-1^)	0.943	< 0.001	0.829–1.000
*k*_*i, multi*_ (s^-1^)	0.876	< 0.001	0.707–1.000
RE^*^ (%)	0.810	< 0.001	0.619–1.000
NLSC^*^	0.850	0.005	0.576–1.000
Anali NoGd	0.800	< 0.001	0.634–0.966
Anali GdAP	0.814	< 0.001	0.644–0.984
Anali GdHBP	0.829	< 0.001	0.681–0.977
MELD	0.857	0.013	0.576–1.000
AOM	0.900	< 0.001	0.699–1.000

*ROC* curves are provided in Supplementary Fig. S3

*Anali GdAP* Anali score based on gadolinium contrast features in the arterial phase, *Anali GdHBP* Anali score based on gadolinium contrast features in the hepatobiliary phase, *Anali NoGd* Anali score derived without gadolinium contrast, *AOM* Amsterdam–Oxford model, *AUC* Area under the curve, *CI* Confidence interval, *k*_*i,multi*_ Hepatocellular uptake rate based on the segmental liver PK model, *k*_*i,single*_ Hepatocellular uptake rate based on the global liver PK model, *MELD* Model for end-stage liver disease, *NLSC* Normalised liver–spleen contrast ratio in the hepatobiliary phase (20 min), *RE* Relative liver enhancement in the hepatobiliary phase (20 min), *ROC* Receiver operated characteristics

^*^ Missing Data for one patient without an endpoint event

### Principal component analysis of segmental hepatobiliary contrast uptake rates

**Fig. 4:**
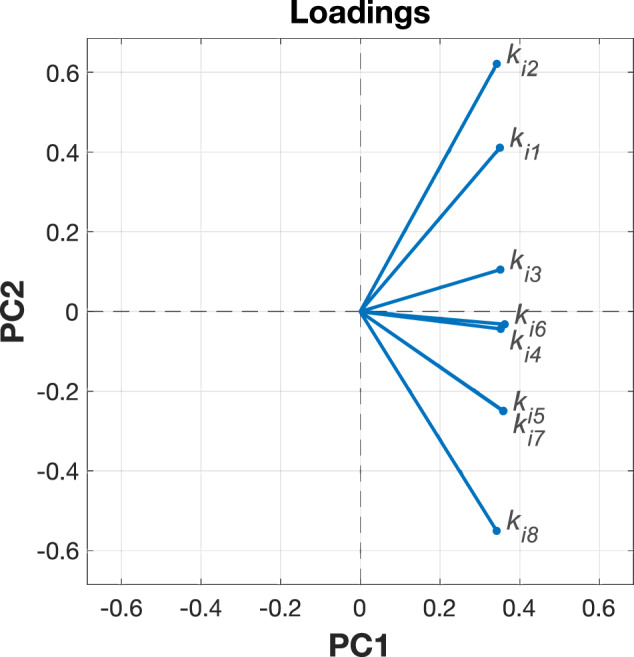
Principal component (PC) analysis loading plot showing the contributions of segmental uptake rates (k_i1_–k_i8_) to the first two principal components (PC1 and PC2). Each dot corresponds to a liver segment and represents its loading on PC1 and PC2. Dots (segments) located close together are positively correlated

Principal component analysis revealed that the first two components accounted for 95% of the total variance (principal component 1: 90.9%, principal component 2: 4.1%). Principal component 1 showed similar positive loadings (the coefficients describing the contribution of each segmental *k*_*i*_ to a component) across all segments (range 0.34–0.36). Principal component 2 showed more variability, with *k*_*i2*_ (0.62) and *k*_*i8*_ (-0.55) having the largest absolute loadings in opposite directions. These patterns are illustrated in the loading plots (Fig. 4).

An exploratory analysis of intrahepatic functional heterogeneity based on the heterogeneity between intrapatient segmental uptake rates is reported in Supplementary Material Section 2—Intrahepatic functional heterogeneity.

## Discussion

This pilot study evaluated a whole-body dynamic PK liver function model based on hepatocellular gadoxetate uptake as a prognostic tool in PSC. The modelled uptake rates demonstrated a strong correlation with the AOM, comparable to MRI-derived and clinical prognostic indices such as Anali scores, RE, and its derived standardised variant, NLSC. The model’s ability to identify patients at risk for adverse events was excellent, comparable to AOM and exceeding MELD, the Analis scores, RE, and NLSC. This finding is particularly encouraging given that the AOM was specifically developed to predict the combined risk of PSC-related death and liver transplantation [16].

These results address the ongoing need for objective, reproducible, and quantitative biomarkers to support prognostication in PSC, which is only partially addressed by current clinical and imaging-based tools [17]. Existing clinical models, such as the Mayo risk score, UK-PSC score, or the AOM show good to excellent accuracy for hepatic decompensation but rely on biochemical markers (*e.g.*, aspartate aminotransferase, alkaline phosphatase, albumin, bilirubin, platelets) that typically change only after significant hepatocellular injury [18] or with advanced fibrosis [19, 20], and thus fail to capture early functional decline.

In contrast, the PK model directly quantifies liver function by modelling the hepatocellular uptake of gadoxetate disodium using dynamic MRI, which is a validated surrogate biomarker of hepatocellular transporter activity [21]. It reflects the activity of organic anion transporting polypeptides 1B1 and 1B3, which are responsible for hepatocellular substrate uptake, mirroring metabolic, detoxification, and drug clearance capacity [22, 23]. This method enables early detection of functional deterioration in progressive liver disease [11, 13, 24].

Our findings suggest that model-derived hepatocellular uptake rates provide prognostic performance comparable to the AOM. Recent evidence showed that the addition of imaging-derived quantitative metrics, such as radiomics, can improve the prognostic performance of AOM and MELD in PSC patients [25]. Similarly, the integration of hepatocellular uptake rates into these existing clinical models could enhance their prognostic performance.

Quantitative MRI-derived biomarkers such as RE have been used to evaluate global and regional liver function and monitor disease progression in PSC [26]. In line with these findings, RE and its derived parameter, NLSC, correlated well with the AOM and MELD in this cohort and showed good discriminative ability for identifying high-risk patients. However, the discriminative performance of the hepatocellular uptake rate trended above that of both RE and NLSC.

Despite their practicality, static indices such as RE or NLSC have inherent methodological limitations. The non-linear relationship between signal intensity and contrast concentration [27] requires empirical correction, typically via reference tissues, which PK models incorporate intrinsically, but which RE does not. The stronger performance of NLSC compared to RE probably reflects this correction step. Moreover, static signal intensity-based measures such as these are highly sensitive to sequence and vendor variability, limiting comparability between examinations and across centres.

In contrast, PK models derive physiologically meaningful parameters from contrast dynamics, producing absolute, reproducible measures of liver function less affected by scanner or sequence variation [11]. Furthermore, static indices such as the contrast enhancement index, which resemble RE, have shown poorer performance than PK model-derived parameters in chronic liver disease assessment [28]. This difference reflects the physiological complexity of gadoxetate, which is distributed between both the extracellular extravascular and hepatocellular compartments. While static indices cannot account for this dual behaviour, PK models explicitly describe the kinetics of contrast agent transport, providing a mechanistic and quantitative assessment of hepatocellular function that static approaches do not achieve.

Finally, PK models are inherently adaptable, allowing progressive refinement, for example, integrating T1 relaxation-based measurements of *k*_*i*_ or incorporating systemic markers of liver function such as bilirubin. This flexibility supports ongoing optimisation and future methodological advancement beyond the limits of static parameters such as RE.

Beyond global assessment, there is merit in regional assessment of hepatic function, especially in heterogeneous diseases such as PSC. As Elkilany et al [26] have highlighted, regional assessment of hepatic function derived from gadoxetate-enhanced MRI can provide valuable prognostic information in PSC, where structural and functional changes are often heterogeneously distributed across the liver [2]. Contrast-enhanced MRI in general has previously been used to calculate both global and regional hepatic function [21, 29–31]. Dynamic contrast-enhanced MRI has also been explored for PK modelling of hepatocellular function, though less so at the regional level. Truhn et al [32], for example, assessed regional liver function using a PK framework but did not specifically evaluate individual Couinaud segments. In contrast, the segmental liver PK model simultaneously estimates contrast concentration and uptake rate for each Couinaud segment, expanding the model from global to regional liver function while preserving the underlying PK model structure. This regional model correlates strongly with the global model and additionally opens opportunities for regional functional assessment of liver function. Such regional models may enable quantitative, mechanistic monitoring of segmental liver function for PSC progression or transplant planning.

Though validated as a prognostic tool for PSC, the Anali scores do suffer from significant interobserver variability [10, 33], which limits their applicability in clinical practice. Being based on morphological changes and qualitative contrast features, the Anali scores can represent a complementary rather than a competing approach to PSC prognostication compared to the model-derived hepatocellular uptake rate. However, recent research combining Anali scores with other quantitative MRI-derived parameters (liver stiffness and splenic volume) has shown that the Anali scores contributed little to the combined multivariable model [34]. It is therefore not unreasonable to assume that combining the Anali scores with the hepatocellular uptake rate would only provide limited gains for the prognostication of PSC patients.

Limitations

The cohort size and limited number of endpoint events represent key limitations, restricting the generalisability of the findings. One of the endpoint events was a case of cholangiocarcinoma, further reducing statistical robustness.

The hepatocellular uptake rate did not correlate with any of the laboratory biomarkers except for a positive correlation with platelet count. This correlation is expected, as previous studies [35, 36] have shown that better liver function is associated with higher platelet counts. However, anticipated correlations with albumin and bilirubin were not observed. The absence of these correlations probably reflects limited sample size and the early disease stage of most participants. In this cohort, markedly elevated bilirubin levels were rare, which may explain the lack of association [37].

Given the limited number of patients and endpoint events, the ROC performance estimates reported in this study should be interpreted as exploratory findings. Validation in larger, independent cohorts will be required to confirm these results.

For the Anali scores, generalisability may have been affected by single-reader assessment, which can introduce bias given the known interobserver variability of this system [7, 10], particularly for score components involving subjective morphological assessment. This limitation should be considered when interpreting comparisons involving Anali-based metrics.

ROI placement was done by consensus review between two experienced abdominal radiologists, which precluded formal assessment of inter-reader reproducibility of the derived PK parameters. Evaluation of reproducibility will therefore be an important aspect of future validation studies aimed at clinical implementation.

Owing to missing relevant clinical data, the Mayo risk score [38] or UK-PSC score [39] could not be calculated, although it would have provided a more disease-specific comparator than MELD for this cohort.

To minimise the risk of model overfitting, the small standard error of the mean values was adjusted to the mean standard error of the mean value of the corresponding time series within each dataset. During training of the segmental liver PK model, a hypothesised standard error of the mean of ±10% of each data point value was applied for the same purpose. These adjustments did not alter the key dynamic behaviour observed in the data. Future studies could enhance model precision by extracting additional ROIs per Couinaud segment to obtain more accurate estimates of data uncertainty. Model fitting was evaluated qualitatively, which was considered sufficient for the study’s purpose. Nevertheless, quantitative assessment, for example, through χ^2^ testing of residuals, may further refine this process. However, the key model parameters, particularly the hepatocellular uptake rate constants, were well identified and robust across all datasets.

In this study, image acquisition extended up to 43 min after contrast agent injection, which in a clinical setting could increase scanner occupancy time and potentially reduce feasibility in routine practice. Forsgren et al [13] demonstrated that reliable model performance can be achieved with acquisition times as short as ten min post-contrast agent injection. This suggests that the data requirements of the PK model are compatible with standard gadoxetate-enhanced MRI protocols currently recommended in clinical guidelines [5, 6].

From a practical perspective, implementing PK modelling would primarily require post-processing software capable of analysing dynamic signal intensity data from routine gadoxetate-enhanced MRI examinations. Integration into clinical workflows would mainly involve automated post-processing and model fitting, rather than major modifications to acquisition protocols. In practice, the PK model benefits from multiple hepatobiliary phase measurements. Inclusion of an additional hepatobiliary phase (for example, at approximately 10 min) may further improve parameter estimation while remaining compatible with routine clinical imaging workflows.

Despite these limitations, the findings highlight the potential of whole-body dynamic gadoxetate-enhanced MRI-based PK modelling to detect early liver function decline for prognostic assessment in PSC. Validation in larger, multicentre cohorts with extended follow-up is warranted to confirm these results and define their broader clinical relevance.

Gadoxetate-enhanced MRI is already widely implemented in the routine evaluation of PSC [5, 6]. Integrating imaging-derived hepatocellular uptake rates as complementary inputs into established biochemical models such as the AOM or UK-PSC score could enhance diagnostic precision, refine risk stratification, and support improved clinical decision-making in PSC management.

In conclusion, this study demonstrates that PK modelling of hepatocellular contrast rates functions as a robust, quantitative biomarker of liver function in PSC. This biomarker performed comparably to established clinical and imaging-based risk scores while providing a direct, objective, and mechanistic assessment of hepatocellular function. The findings support the notion that dynamic, model-derived biomarkers yield valuable prognostic information that can be used to complement existing prognostic tools. Integrating such quantitative metrics into established clinical scoring systems, such as AOM or the UK-PSC model, may facilitate earlier identification of patients at risk of poor prognosis and adverse outcomes.

## Supplementary information

**Additional File 1:** **Fig. S1** Example of model agreement between model simulation and gadoxetate time-series data for a patient for a single examination (U1) for the segmental liver pharmacokinetic model. The model can find satisfactory agreement with data from all segments as well as data from the spleen. The model was trained to the data from each examination (and patient) separately. The grey area indicates the time 0 to 3 min, where data was not used for model training. **Fig. S2** Example of model agreement between model simulation and gadoxetate time-series data for a patient for a single examination (U1) for the global liver pharmacokinetic model. **Fig. S3** ROC analysis. 95% confidence intervals are given in parentheses after each AUC value. *AOM* Amsterdam–Oxford model, *AUC* Area under the curve, *Ki,single* Hepatocellular uptake rate based on the global liver pharmacokinetic model, *NLSC* Normalised liver-spleen contrast ratio. *ROC* Receiver Operating characteristic. **Table S1** Correlation of prognostic parameters with laboratory data, clinical scores, and endpoint events. **Table S2** Sensitivity, specificity, PPV, and NPVof *Ki,single*, AOM, NLSC, and RE.

## Data Availability

The datasets generated and/or analysed during the current study are not publicly available due to strict Swedish patient data integrity laws, but are available from the corresponding author on reasonable request.

## Abbreviations

AOM: Amsterdam–Oxford model
AUC: Area under the (receiver operating characteristic) curve
Gd-EOB-DTPA: Gadolinium-ethoxybenzyl-diethylenetriamine pentaacetic acid
MELD: Model for end-stage liver disease
MRI: Magnetic resonance imaging
NLSC: Normalised liver-spleen contrast ratio
PK: Pharmacokinetic
PSC: Primary sclerosing cholangitis
RE: Relative enhancement
ROC: Receiver operating characteristic
ROI: Region of interest
