# Supplementary material for: Pharmacokinetic modelling of MRI-based liver function for risk assessment in primary sclerosing cholangitis: a prospective pilot study
Source: Eur Radiol Exp. 2026 Jun 18;10:91. doi: 10.1186/s41747-026-00764-5 (PMC13280068; doi:10.1186/s41747-026-00764-5)
Supplement: Supplementary file 1 — Additional File 1: Fig. S1 Example of model agreement between model simulation and gadoxetate time-series data for a patient for a single examination (U1) for the segmental liver pharmacokinetic model. The model can find satisfactory agreement with data from all segments as well as data from the spleen. The model was trained to the data from each examination (and patient) separately. The grey area indicates the time 0 to 3 min, where data was not used for model training. Fig. S2 Example of model agreement between model simulation and gadoxetate time-series data for a patient for a single examination (U1) for the global liver pharmacokinetic model. Fig. S3 ROC analysis. 95% confidence intervals are given in parentheses after each AUC value. AOM Amsterdam–Oxford model, AUC Area under the curve, Ki,single Hepatocellular uptake rate based on the global liver pharmacokinetic model, NLSC Normalised liver-spleen contrast ratio. ROC Receiver Operating characteristic. Table S1 Correlation of prognostic parameters with laboratory data, clinical scores, and endpoint events. Table S2 Sensitivity, specificity, PPV, and NPVof Ki,single, AOM, NLSC, and RE. [file 41747_2026_764_MOESM1_ESM.pdf]

Pharmacokinetic Modelling of MRI-based Liver Function for Risk Assessment in Primary Sclerosing Cholangitis: A Prospective Pilot Study

ELECTRONIC SUPPLEMENTARY MATERIAL

Section 1 – Mathematical modelling

The modelling approach has previously been described [1, 2]. Briefly, all presented models were based on ordinary differential equations and they were implemented in MATLAB (2022a, MathWorks, Natick, MA, USA) using the SB Toolbox2 package [3]. For model parameter estimation the enhanced scatter search algorithm from the MEIGO toolbox was used [4]. The parameter estimation was performed separately for each patient and examination combination.

The Pharmacokinetic model of hepatic Couinaud segments

We constructed a composite regional pharmacokinetic model by extending the previously published global model (1, 2). The regional model describes each of the Couinaud segments (4a and 4b, were approximated to a single compartment) as separate functional units. In practice, the model was constructed by repeating the equations governing the hepatic Gd-EOB-DTPA in the global model, for each segment. Furthermore, model parameters were added for each segment, governing the influx and efflux rates, as well as the segmental volumes (approximated to total volume of 1.54L, with segmental sizes describe by Mise et al. (5)). In general, the regional model follows the same structure as the previously published global model but extends the number of separate hepatic compartments.

The following section will go through the model equations. Just as the global model, the *central model compartment* describes the Gd-EOB-DTPA present in the plasma, and is denoted C_p, and has the following derivative:

$$\frac{d}{dt}C_p = \frac{r_{\mathrm{ECS}} \cdot \mathrm{V_{ECS}} + u - r_h - r_{urine}}{V_b \cdot (1 - \mathrm{Hct})}, C_p(0) = 0 \qquad \textbf{(Eq. 1)}$$

where, r_{ECS} (Eq. 2) is the model reaction describing fluxes to extra cellular space (ECS), the constant $\mathrm{V_{EES}}$ which is the ECS volume assumed to be 14.77 L (6), *u* is the model input (injection of Gd), r_h (Eq. 3) is the model reaction describing the uptake from all eight segments, r_{urine} is the reaction describing the removal via urine, the blood plasma volume constant $V_b$assumed to be 2.57 L (6), and the hematocrit (Hct).

The derivate also includes the reaction r_{ECS} which represents diffusion and is written as:

$$r_{ECS} = k_d \cdot (C_{ECS} - C_p \cdot \mathrm{Alb}) \qquad \textbf{(Eq. 2)}$$

where, C_{ECS} (Eq. 9) is the model state describing Gd-EOB-DTPA in the ECS compartment, C_p is the plasma compartment, $k_d$is the rate parameter governing the diffusion between the two compartments, and Alb is a constant which is represents the fraction of Gd-EOB-DTPA not bound to albumin and had a value of 0.9.

The next reaction is the renal clearance, r_{urine} which is written as:

$$r_{urine} =\cdot\ C_p \cdot \mathrm{Alb}\ k_{CLr} \qquad \textbf{(Eq. 3)}$$

where, k_{CLr} is the rate parameter for renal clearance set to 0.001845 s^{-1}.

The next reaction is $r_h$which describes the uptake of Gd-EOB-DTPA into each hepatic segment which is written as:

$$r_h = \sum_j^{\mathbb{N}} r_{h_j} = \left(\sum_j^{\mathbb{N}} k_{i_j} \cdot \mathrm{V_{S_j}} \right) \cdot F_h \cdot C_p \cdot Alb \qquad \textbf{(Eq. 4)}$$

$$\mathbb{N} = \forall\, [1,..,8]$$

where, r_{h_j} is the uptake for the segment with index j, N is a set of numbers between 1 to 8 which represent the index for all segments which exist based the patient and examination data, k_{i_j} is the model rate parameter for the Gd-EOB-DTPA uptake for each segment, $\mathrm{V_{S_j}}$ is the specific segment volume, and Fh was set to be 0.68 (6) and is the constant describing the ratio of hepatocytes in liver (compared to other tissue).

Lastly, for the C_p derivative we also need to describe the model input, u, which represents the injection of Gd-EOB-DTPA into plasma during the MRI examination.

The input variable can be written as:

$$u = C_{EOB} \cdot \mathrm{k_{syr}} \qquad \textbf{(Eq. 5)}$$

where C_{EOB} is the concentration of Gd-EOB-DTPA in the syringe and $\mathrm{k_{syr}}$ (s^{-1}) is the input injection rate. To turn the input on and off at the correct dosage the state, C_{EOB} is used in combination with the N_{dose} state.

The C_{EOB} have a derivative of zero and can be written

$$\frac{d}{dt} C_{EOB} = 0, C_{EOB}(0) = 0.25\ \mu\mathrm{mol} \qquad \textbf{(Eq. 6)}$$

$$C_{EOB}(t) = \begin{cases} 0.25\ N_{dose} < dose \\ 0\ N_{dose} \geq dose \end{cases}$$

where $N_{dose}$is the model state for the dosage of Gd-EOB-DTPA and the *dose* parameter is the dose administered to the patient.

The N_{dose} derivative can be written as:

$$\frac{d}{dt} N_{dose} = C_{EOB} \cdot \mathrm{k_{syr}}, N_{dose}(0) = 0 \qquad \textbf{(Eq. 7)}$$

$$dose = 25\ (umol \cdot kg^{-1}) \cdot \mathrm{BW\ (kg)}$$

This concluded description of the $C_p$derivative.

The next part of the model is the concentration of Gd-EOB-DTPA in the hepatocytes in each segment. This is represented by the model states $C_{h_j}$with *j* being the index for each segments, each having a derivative written in the following general form:

$$\frac{d}{dt} C_{h_j} = r_{h_j} - k_{e_j} C_{h_j}\ , C_{h_j}(0) = 0 \qquad \textbf{(Eq. 8)}$$

where, the first term r_{h_j} is the segment-specific uptake (described above), and the second term is the segment-specific efflux to bile. Each efflux is governed by a rate parameter k_{e_j}. (s^{-1}).

The last part of the model describes the Gd-EOB-DTPA present in the ECS and is written as:

$$\frac{d}{dt} C_{ECS} = -r_{ECS}, C_{ECS}(0) \qquad \textbf{(Eq. 9)}$$

where r_{ECS} is the diffusion term between plasma and ECS preciously described in Eq. 2.

To make the model output comparable with the image-data we have previously devised measurement equation accounting for Gd-EOB-DTPA in either the hepatocytes, plasma or ECS in the image ROI (1, 2).

The first is the concentration of Gd-EOB-DTPA in the liver, and here for each segment *j* the equation can be written as:

$$y_{segment_j} = C_{h_j} \cdot \mathrm{F_h} + \left(C_p \cdot \mathrm{F_b} \cdot (1 - Hct) + C_{ECS} \cdot F_{lees} \right) \cdot \frac{\mathrm{V_{S_j}}}{V_{total}}\ j = \mathbb{N} \qquad \textbf{(Eq. 10)}$$

where all parameters and states have been previously defined except; $\mathrm{F_b}$which is the fraction of blood in the liver and has a value of 0.12 (6), Hct is the hematocrit constant and has a value of 0.43 (6), and F_{lees} is the ECS fraction in the liver and has a value of 0.20 (6), and lastly V_{total} is the total liver volume.

The next output is the equation for Gd-EOB-DTPA in the spleen, defined as:

$$y_{spleen} = \quad C_p \cdot \mathrm{F_s} \cdot (1 - Hct) + \; C_{ECS} \cdot F_{sees}, \quad \textbf{(Eq. 11)}$$

where $\mathrm{F_s}$ is the fraction of spleen volume with blood, and F_{sees} is the fraction of ECS in the spleen volume.
This completes the description of the model equation.

To summarize, we have extended our previously published model (1, 2) to include a description of each Couinaud segment as separate functional units.

Examples of pharmacokinetic model agreement to data
In this section we present an example of model agreement using the segmental liver pharmacokinetic model and the global liver pharmacokinetic model. In the figures, the solid line is the model simulation (the measurement equation Eq. 10 for liver or global equivalent, or Eq. 11 for spleen) and the error bars are the measured data (for segment data the standard error of the mean was set to ±10% from the measurement values).

Supplementary references

1. Forsgren MF, Dahlqvist Leinhard O, Dahlstrom N, Cedersund G, Lundberg P (2014) Physiologically realistic and validated mathematical liver model reveals hepatobiliary transfer rates for Gd-EOB-DTPA using human DCE-MRI data. PLoS One 9:e95700. https://doi.org/10.1371/journal.pone.0095700

2. Forsgren MF, Karlsson M, Dahlqvist Leinhard O et al (2019) Model-inferred mechanisms of liver function from magnetic resonance imaging data: validation and variation across a clinically relevant cohort. PLoS Comput Biol 15:e1007157. https://doi.org/10.1371/journal.pcbi.1007157

3. Schmidt H, Jirstrand M (2006) Systems biology toolbox for MATLAB: a computational platform for research in systems biology. Bioinformatics 22:514–5. https://doi.org/10.1093/bioinformatics/bti799

4. Egea JA, Henriques D, Cokelaer T et al (2014) MEIGO: an open-source software suite based on metaheuristics for global optimization in systems biology and bioinformatics. BMC Bioinformatics 15:136. https://doi.org/10.1186/1471-2105-15-136

5. Mise Y, Satou S, Shindoh J et al (2014) Three-dimensional volumetry in 107 normal livers reveals clinically relevant inter-segment variation in size. HPB 16:439–447. https://doi.org/10.1111/hpb.12157

6. Levitt DG (2003) The pharmacokinetics of the interstitial space in humans. BMC Clin Pharmacol 3:3. https://doi.org/10.1186/1472-6904-3-3

Supplementary figures

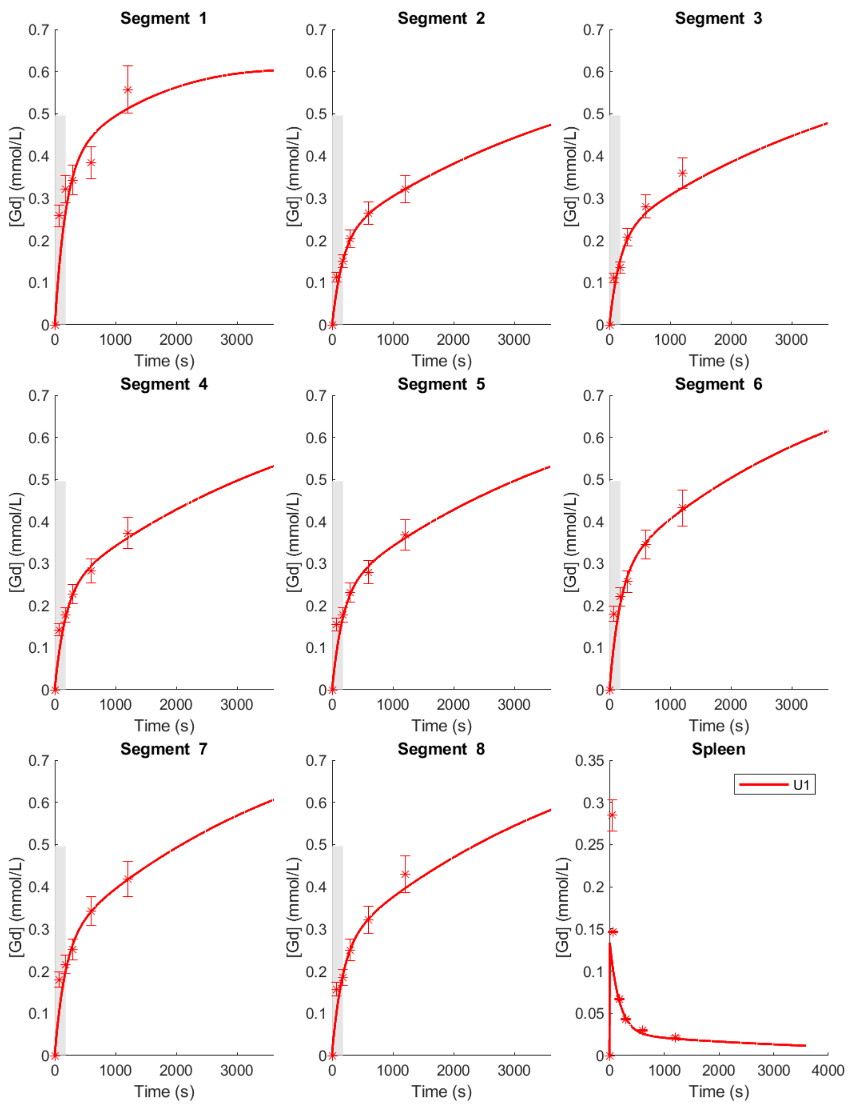

Fig. S1 Example of model agreement between model simulation and gadoxetate time-series data for a patient for a single examination (U1) for the segmental liver pharmacokinetic model. The model can find satisfactory agreement to data from all segments as well as data from the spleen. The model was trained to the data from each examination (and patient) separately. The grey area indicates the time 0 to 3 min where data was not used for model training.

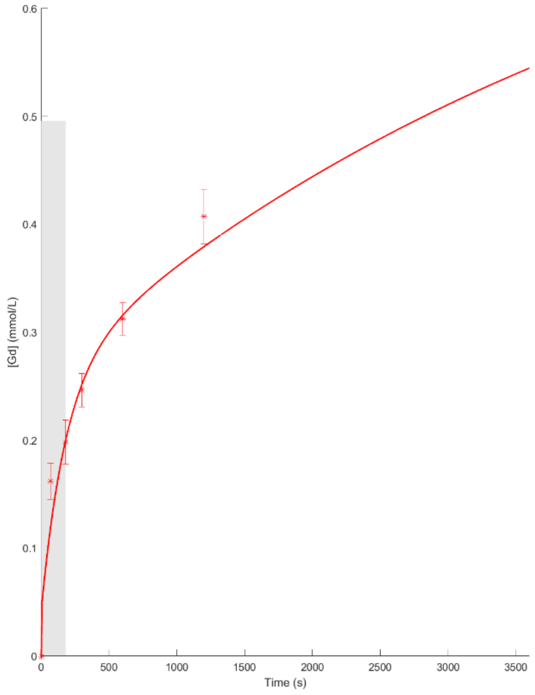

Fig. S2 Example of model agreement between model simulation and gadoxetate time-series data for a patient for a single examination (U1) for the global liver pharmacokinetic model.

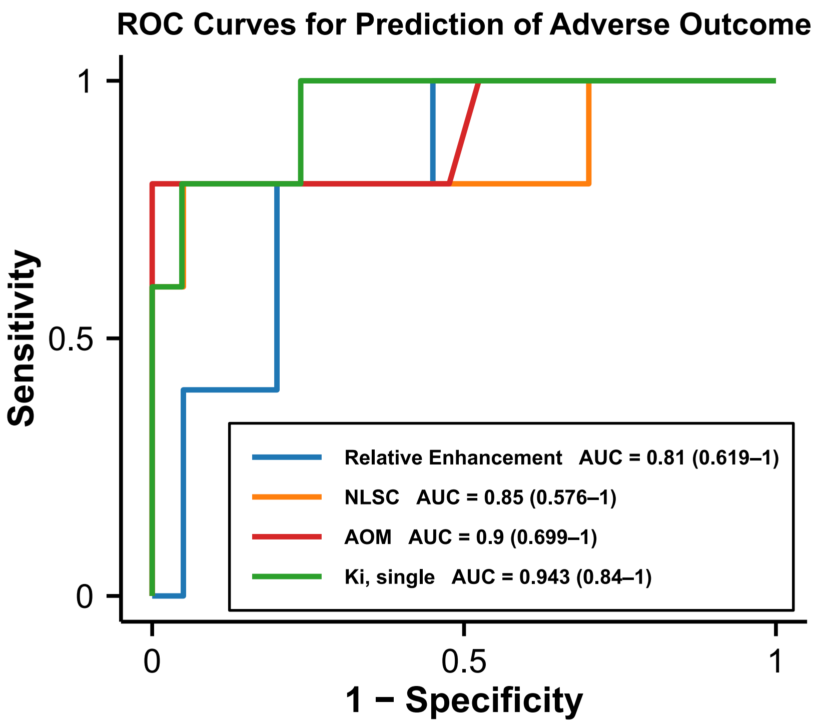

Fig. S3 ROC analysis. 95% confidence intervals are given in parentheses after each AUC value. *AOM* Amsterdam-Oxford model, *AUC* Area under the curve, $K_{i,single}$ Hepatocellular uptake rate based on the global liver pharmacokinetic model, *NLSC* Normalized liver-spleen contrast ratio. *ROC* Receiver Operating characteristic.

Supplementary tables

Table S1 Correlation of prognostic parameters with laboratory data, clinical scores, and endpoint events

Parameter	Anali NoGd (ρ/*p*-*value*)	Anali GdAP (ρ/*p*-*value*)	Anali GdHBP (ρ/*p*-*value*)	$k_{i,single}$ (ρ/*p*-*value*)	$k_{i, multi}$ (ρ/*p*-*value*)	RE (ρ/*p*-*value*)*	NLSC (ρ/*p*-*value*)*
ALT	0.306 / 0.129	0.073 / 0.723	0.200/ 0.328	-0.100 / 0.626	-0.129 / 0.529	0.139 / 0.506	-0.254 / 0.221
AST	0.280 / 0.175	-0.001 / 0.998	0.229 / 0.271	-0.081 / 0.699	-0.078 / 0.710	0.076 / 0.724	-0.295 / 0.162
γGT	**0.446 / 0.022**	0.239 / 0.240	0.283 / 0.161	-0.067 / 0.744	-0.088 / 0.670	0.028 / 0.892	**-0.426 / 0.034**
Total serum bilirubin	**0.566 / 0.003**	0.316 / 0.116	**0.487 / 0.012**	-0.358 / 0.072	-0.273 / 0.178	**-0.618 / <0.001**	**-0.706 / <0.001**
Albumin	0.089 / 0.664	-0.125 / 0.544	-0.034 / 0.868	0.358 / 0.072	0.276 / 0.172	0.324 / 0.114	-0.015 / 0.945
Platelets	-0.280 / 0.166	**-0.405 / 0.040**	-0.388 / 0.051	**0.614 / 0.001**	**0.489 / 0.012**	**0.525 / 0.008**	**0.529 / 0.008**
Creatinine	-0.298 / 0.140	-0.372 / 0.061	-0.384 / 0.053	0.118 / 0.567	-0.041 / 0.842	**0.421 / 0.036**	0.156 / 0.457
INR	0.359 / 0.072	**0.403 / 0.041**	0.347 / 0.082	-0.308 / 0.126	-0.217 / 0.287	-0.383 / 0.059	-0.312 / 0.134
MELD	**0.594 / 0.001**	0.321 / 0.110	**0.419 / 0.033**	**-0.429 / 0.029**	**-0.393 / 0.047**	**-0.497 / 0.011**	**-0.714 / <0.001**
AOM	**0.490 / 0.011**	**0.532 / 0.005**	**0.504 / 0.009**	**-0.557 / 0.003**	**-0.490 / 0.011**	**-0.585 / 0.002**	**-0.639 / <0.001**
Endpoint event	**0.417 / 0.034**	**0.460 / 0.018**	**0.478 / 0.014**	**-0.605 / 0.001**	**-0.514 / 0.007**	**-0.430 / 0.032**	**-0.485 / 0.014**

All values are Spearman correlation coefficients (ρ) / *p*-values. Significant correlations are marked in bold. *ALP* Alkaline phosphatase, *ALT* Alanine transaminase, *Anali GdAP* Anali score based on gadolinium contrast features in the arterial phase, *Anali GdHBP* Anali score based on gadolinium contrast features in the hepatobiliary phase, *Anali NoGd* Anali score derived without gadolinium contrast, *AOM* Amsterdam–Oxford model, *AST* Aspartate transaminase, *γGT* Gamma-glutamyl transferase, *INR* International normalized ratio, *MELD* Model for end-stage liver disease, *NLSC* Normalized liver–spleen contrast ratio in the hepatobiliary phase (20 min), *RE* Relative liver enhancement in the hepatobiliary phase (20 min), $k_{i,single}$ Hepatocellular uptake rate based on the global liver pharmacokinetic model, $k_{i,multi}$ Hepatocellular uptake rate based on the segmental liver pharmacokinetic model. * Missing data: Data from one patient without endpoint event missing

Table S2 Sensitivity, specificity, PPV, and NPVof $K_{i,single}$, AOM, NLSC, and RE

Biomarker	Threshold	Sensitivity	Specificity	PPV	NPV
$K_{i,single}$ (s^{-1})	0.002	1.000	0.762	0.500	1.000
AOM	2.615	0.800	1.000	1.000	0.955
NLSC	1.300	0.800	0.950	0.800	0.950
RE (%)	67.716	0.800	0.800	0.500	0.941

Thresholds were calculated based on Youden's index. *AOM* Amsterdam–Oxford model, *AUC* Area under the curve, $k_{i,single}$ Hepatocellular uptake rate based on the global liver pharmacokinetic model, *NLSC* Normalized liver–spleen contrast ratio in the hepatobiliary phase (20 min), *NPV* Negative predictive value, *PPV* Positive predictive value, *RE* Relative liver enhancement in the hepatobiliary phase (20 min).

Section 2 – Intrahepatic functional heterogeneity

To explore whether intrahepatic heterogeneity of hepatocellular function is associated with clinical outcomes, an exploratory analysis of segmental functional variability was performed using the outputs of the segmental liver PK model.

For each patient, the hepatocellular uptake rated for all eight Couinaud liver segments (k_{i1}–k_{i8}) from the last available MRI examination was estimated. Intrahepatic functional heterogeneity was quantified using the coefficient of variation (CoV), defined as the standard deviation (SD) divided by the mean of the segmental uptake rates k_i (SD/mean). CoV was selected as a scale-independent heterogeneity metric, as the SD of segmental uptake rates showed a strong positive correlation with the mean uptake rate (Spearman $\rho = 0.85$), whereas CoV demonstrated only a weak correlation with the mean ($\rho = -0.22$).

Segmental CoV tended to be higher in patients who experienced adverse endpoint events compared to those who did not (median CoV 20% compared to 10%), although this difference did not reach statistical significance (Mann-Whitney *U* test, $p = 0.157$). Coefficient of variation showed a borderline positive correlation with MELD score (Spearman $\rho = 0.377$, $p = 0.057$). At receiver operating characteristic analysis, CoV showed a moderate discriminative performance for endpoint events (AUC = 0.71), with wide confidence intervals reflecting the limited number of events (95% CI: 0.35–1.00).

Overall, while no statistically significant associations were observed in this pilot cohort, these exploratory findings suggest that increased intrahepatic functional heterogeneity may be associated with more advanced disease and adverse outcomes. Given the limited sample size and number of endpoint events, these results should be interpreted cautiously and warrant further investigation in larger multicenter cohorts.
